# Ion Transport Properties and Ionicity of 1,3-Dimethyl-1,2,3-Triazolium Salts with Fluorinated Anions

**DOI:** 10.3390/ma11091723

**Published:** 2018-09-14

**Authors:** Martin Pulst, Yury Golitsyn, Detlef Reichert, Jörg Kressler

**Affiliations:** Faculty of Natural Sciences II, Martin Luther University Halle-Wittenberg, D-06099 Halle (Saale), Germany; martin.pulst@chemie.uni-halle.de (M.P.); yury.golitsyn@physik.uni-halle.de (Y.G.); detlef.reichert@physik.uni-halle.de (D.R.)

**Keywords:** 1,2,3-triazolium salts, ionic liquids, ionicity, impedance spectroscopy, ion conductivity, diffusion coefficients, viscosity

## Abstract

1,2,3-Triazolium salts are an important class of materials with a plethora of sophisticated applications. A series of three novel 1,3-dimethyl-1,2,3-triazolium salts with fluorine, containing anions of various size, is synthesized by methylation of 1,2,3-triazole. Their ion conductivity is measured by impedance spectroscopy, and the corresponding ionicities are determined by diffusion coefficients obtained from ^1^H and ^19^F pulsed field gradient nuclear magnetic resonance (PFG NMR) spectroscopy data, revealing that the anion strongly influences their ion conductive properties. Since the molar ion conductivities and ionicities of the 1,3-dimethyl-1,2,3-triazolium salts are enhanced in comparison to other 1,2,3-triazolium salts with longer alkyl substituents, they are promising candidates for applications as electrolytes in electrochemical devices.

## 1. Introduction

Ionic liquids (ILs) are organic salts with a melting temperature below 100 °C that have aimed at facilitating green and sustainable chemistry through an attractive combination of their unique properties such as nontoxicity, nonflammability, high ion conductivity, negligible vapor pressure, excellent solvating properties, and good thermal stability [1]. Imidazolium salts are the golden standard at the moment [2] but 1,2,3-triazolium salts (TRSs) are a class of ILs which receive continuously increasing interest due to their facile accessibility via Cu(I)-catalyzed Huisgen-type 1,3-dipolar cycloaddition (CuAAC, “click” reaction), and subsequent *N*-alkylation in quantitative yields [1,3,4,5,6]. Optionally, the anion can be exchanged in an additional so-called salt metathesis step [1], which enables different combinations of cations and anions for TRSs with tuneable properties for a plethora of applications as reaction media [7,8], antifungal agents [9,10], molecular machines [11,12], sensors [13], anticancer drugs [14], precursors for *N*-heterocyclic carbene ligands [15,16], or electrolyte systems in electrochemical devices such as batteries, fuel cells, transistors, and solar cells [17,18,19,20].

A fundamental understanding of charge transport in ILs is of tremendous importance for basic scientific research as well as their applications as electrolytes. Because of their strong ionic character, the degree of dissociation of ILs is defined as *α* = 1 [21] but not all ions contribute identically to the conduction process. On the one hand, the presence of strong intermolecular dispersion and repulsion forces, as well as Coulomb interactions in the salt melt, cause the formation of aggregates, which contribute less to the conductivity as compared to free ions [22,23]. On the other hand, the charge transfer might also take place between the cations and anions, which reduces their total charges [24]. In order to quantify the fraction of the mobile charge carriers, the ionicity *I* is introduced as the number of mobile charge carriers divided by the total number of ions to describe the deviation of the measured ion conductivity with the corresponding value calculated from diffusion or viscosity data [21,22]. The typical values of *I* range between 0.4 ≤ *I* ≤ 0.8 depending on the molecular structure of the IL; i.e., 20–60% of the ions do usually not contribute to the conduction process [21,22,23,24,25,26,27]. Obviously, the highest ionicities are obtained when the cations have only short alkyl substituents, while the value of *I* decreases with an increasing number of methylene groups, as revealed for imidazolium [21] and 1,2,4-triazolium salts [28]. Thus, one would expect that their structure analogous 1,3-dimethyl-1,2,3-triazolium salts would be the best ion conductors having the highest ionicities of all TRSs but they have not yet been studied with respect to their ion conductivity properties, since CuAAC is not well suited for their syntheses, which require reactions of potentially explosive methyl azide with a high shock sensitivity [29]. Furthermore, the difficult purification after the “click” reaction, i.e., the complete removal of reactants and the used copper catalyst, are, if at all, only possible with great effort [1,17]. Obviously, this might be the reason that the values of *I* are only calculated for 1,2,3-triazolium salts with propyl substituents (0.53 ≤ *I* ≤ 0.63) [25], but that their ionicities are lower than the respective values known for the best imidazolium salts (*I* = 0.76) [21]. Thus, there is still a strong demand for the development of new 1,2,3-triazolium salts that are able to compete with the excellent performances of their imidazolium analogues.

In this study, we report the synthesis of three novel 1,3-dimethyl-1,2,3-triazolium salts bearing fluoride, triflate and bis(trifluoromethane sulfon)imide anions, respectively. Impedance spectroscopy (IS), as well as ^1^H and ^19^F pulsed field gradient nuclear magnetic resonance (PFG NMR) spectroscopy are employed in order to obtain an in-depth understanding of the relationship between the ion transport properties and the molecular structure of the 1,2,3-triazolium salts under investigation.

## 2. Materials and Methods

### 2.1. Materials

**Figure 1:**
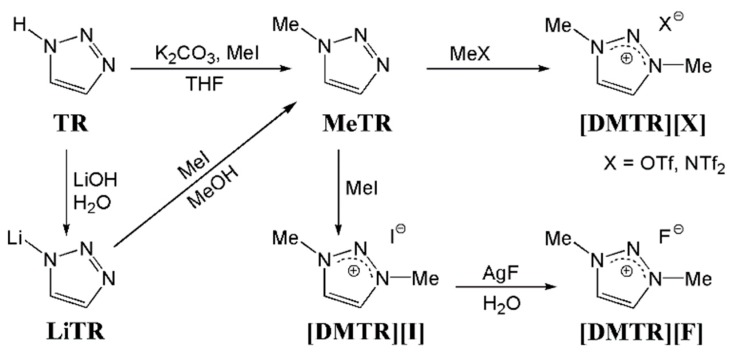
Synthesis of the 1,3,-dimethyl-1,2,3-triazolium salts.

The 1,3-dimethyl-1,2,3-triazolium salts were synthesized by direct methylation of 1,2,3-triazole or via the intermediate lithium 1,2,3-triazolate (see Figure 1). The detailed synthesis procedures and extensive characterization data of all the reported 1,2,3-triazolium salts can be found in the Supplementary Materials. 1,2,3-Triazole used for their syntheses was received from abcr GmbH (Karlsruhe, Germany). Methyl iodide, potassium carbonate, *N*-methyl bis((trifluoromethyl)sulfonyl)imide, and methyl triflate were purchased from Sigma-Aldrich/Merck (Darmstadt, Germany). Silver fluoride was received from Alfa Aesar (Haverhill, MA, USA). Tetrahydrofuran (THF), dimethyl sulfoxide (DMSO), methylene chloride, and methanol were purchased from VWR (Radnor, PA, USA), Carl Roth (Karlsruhe, Germany), Overlack (Mönchengladbach, Germany), and Brenntag (Essen, Germany), respectively.

### 2.2. Methods

The ion conductivity of the 1,3-dimethyl-1,2,3-triazolium salts was measured with a Broadband Dielectric Spectrometer alpha series from novocontrol GmbH. All experiments were performed under the flow of dry nitrogen gas in the frequency range of 10^−1^ Hz ≤ *ν* ≤ 10^7^ Hz. The samples were placed between two gold plated electrodes having a diameter of 20 mm, and a fixed distance of 100 μm. After cooling the triazolium salts to *T* = −20 °C, the data was recorded in temperature steps of *ΔT* = 5 K upon heating.

^1^H and ^19^F pulsed field gradient (PFG) NMR spectroscopy was carried out for diffusion coefficient measurements. The spectra of the molten 1,3-dimethyl-1,2,3-triazolium salts were recorded with a Bruker Avance II 400 MHz instrument between 50 °C ≤ *T* ≤ 90 °C in steps of *ΔT* = 5 K. A stimulated echo with bipolar gradient (STEBP) was used as sequence with a gradient time of *δ* = 2 ms and a varying diffusion times *Δ* of 20–80 ms, depending on the nucleus and the measurement temperature.

The densities of the molten 1,3-dimethyl-1,2,3-triazolium salts were measured with an Anton Paar DMA 60 density meter equipped with a DMA 602 measuring cell in the temperature range of 50 °C ≤ *T* ≤ 90 °C in steps of *ΔT* = 5 K. A thermostat from JULABO GmbH controlled the temperature during the measurement, and the ILs were allowed to equilibrate 5 min before the measurement at each temperature step.

An Anton Paar Physica MCR 301 shear rheometer equipped with a CP25-2/TG measurement system having a cone-plate geometry with a diameter of 25 mm, an angle of 2°, and a gap of 51 μm recorded the viscosities of the molten triazolium salts. The values were measured in the temperature range of 50 °C ≤ *T* ≤ 90 °C in the steps of *ΔT* = 5 K, at different shear rates (10^–2^ Hz ≤ $\dot{\gamma }$ ≤ 10^2^ Hz).

Thermogravimetric analysis (TGA) was performed under continuous nitrogen flow with a heating rate of 10 K min^−1^ in the temperature range of 25 °C ≤ *T* ≤ 800 °C using a Mettler Toledo TGA/SDTA 851^e^ module. Alumina pans were filled with about 5−15 mg of sample for the measurement.

Differential scanning calorimetry (DSC) was performed under continuous nitrogen flow using a Mettler Toledo DSC 822^e^ module. Aluminum pans were filled with about 5−15 mg of sample and the DSC traces were recorded in the temperature range of −40 ≤ *T* ≤ 150 °C with a heating rate of 5 K min^−1^.

NMR spectroscopy was used to confirm the structure and purity of the newly synthesized triazolium salts. The measurements were performed with a magritek Spinsolve SPA550 43 MHz benchtop NMR spectrometer. The deuterated methanol was purchased from ARMAR Chemicals. The calibration of the NMR scale of the ^1^H chemical shifts followed the signal of the residual solvent (CD_3_OD: $\delta_{1_{H}}$ = 4.87 ppm, $\delta_{1_{H}}$ = 3.31 ppm) to TMS. The ^19^F NMR spectra were referenced with an internal Teflon standard (broad signal at $\delta_{19_{F}}$ ≈ −72 ppm) to CFCl_3_.

## 3. Results and Discussion

**Figure 2:**
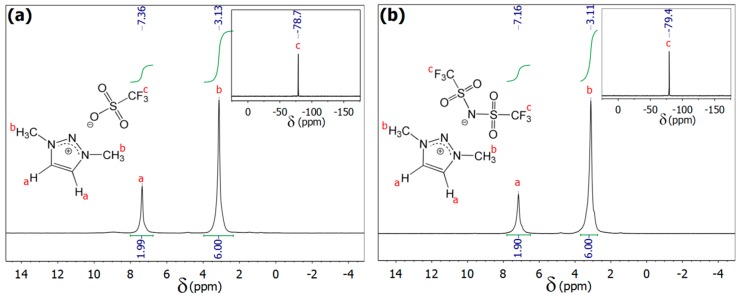
Solvent-free ^1^H NMR spectra (43 MHz) of molten (**a**) [DMTR][OTf] and (**b**) [DMTR][NTf_2_] with assignment of the resonances. The insets show the respective solvent-free ^19^F NMR spectra (41 MHz).

We synthesized a series of three 1,3-dimethyl-1,2,3-triazolium salts by direct methylation of 1,2,3-triazole (TR) or via the intermediate lithium 1,2,3-triazolate (LiTR, see Figure 1). Obviously, this approach did not contain the highly hazardous methyl azide as necessary for the commonly applied CuAAC. The SN_2_ functionalization of the commercially available 1,2,3-triazole or its lithiated derivative is more suitable since they are usually synthesized via alternative azide-free synthesis routes by the supplier [30,31,32,33]. Thus, it has already been considered for the preparation of TRSs in the groups of Begtrup [29] and Coughlin [17]. Furthermore, the products are obtained in good yields which is also interesting for potential industrial applications. The structure and purity of the synthesized TRSs was confirmed by unambiguous assignment of all the resonances of the ^1^H, ^13^C, and ^19^F NMR spectra, as exemplarily shown in Figure 2. The other NMR spectra are depicted in the Supplementary Materials, Figures S1–S6. The cation of the three TRSs under investigation has deliberately not been varied, but three fluorine containing anions are selected (cf. Table 1), which have totally different ion volumes. While the 1,3-dimethyl-1,2,3-triazolium fluoride ([DMTR][F]) bears the smallest anion (*V^−^* = 10 Å^3^), there are also two more TRSs with the medium-sized triflate ([OTf]^−^) and the large bis(trifluoromethane sulfon)imide ([NTf_2_]^−^) anion having volumes of *V^−^* = 129 Å^3^ and *V^−^* = 248 Å^3^, respectively [34,35,36].

**Figure 3:**
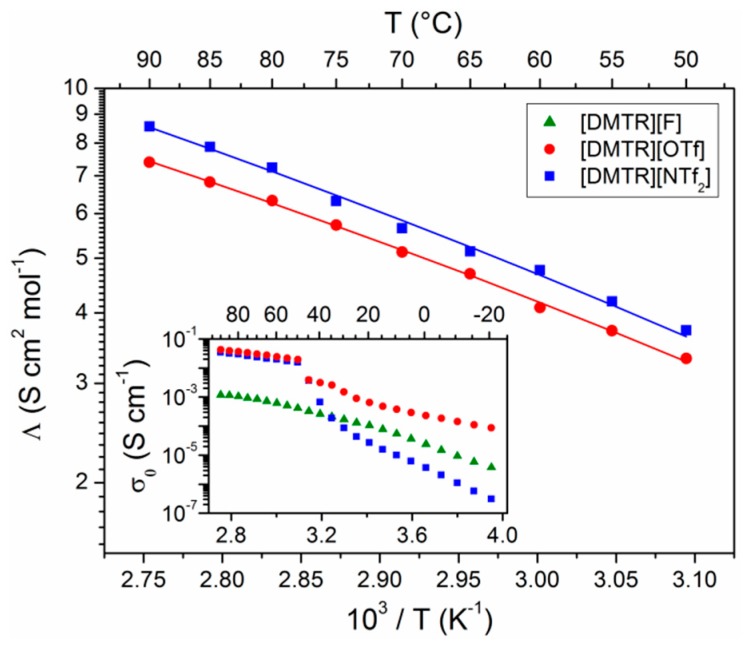
Molar ion conductivities of [DMTR][OTf] and [DMTR][NTf_2_] as functions of the inverse temperature. The lines are the best fits with the Vogel–Fulcher–Tammann (VFT) equation. The inset shows the ion conductivity of all three 1,3-dimethyl-1,2,3-triazolium salts under investigation in an extended temperature range.

The three TRSs were investigated by impedance spectroscopy (IS) over a broad frequency and temperature range. The complex conductivity σ* = σ*’ + i*σ*”* is determined from the measured impedance Z* (σ* = d/(A × Z*), where *d* and *A* are the distance between the two electrodes and the interfacial area between an electrode and the electrolyte, respectively). An analysis of the real part of the complex conductivity σ*’* as a function of the angular frequency *ω* with the Dyre equation [37] yields the ion conductivity σ*_0_*, which is shown as function of the inverse temperature in the inset of Figure 3. While the temperature-dependent conductivity of crystalline [DMTR][F] shows a continuous behavior in the measured range, whereas a transition is observed in the respective curves of [DMTR][OTf] and [DMTR][NTf_2_], which are related to their melting points at *T_m_* = 49.0 °C and *T_m_* = 46.1 °C, respectively (see Table 1 and Figure S12). Thus, the molar ion conductivity *Λ* of these two ILs can be calculated in the molten state using their densities *ρ* (cf. Figure S13), and the respective values of the molar mass (*Λ = σ_0_ × M/ρ*). Figure 3 shows the temperature-dependent molar ion conductivity of [DMTR][OTf] and [DMTR][NTf_2_], which can be described with the Vogel–Fulcher–Tammann (VFT) equation by [18,38]:

(1) $$\Lambda =\Lambda_{\infty }\;e^{-\frac{E_{a}}{R\cdot \left(T-T_{0}\right)}}$$

where *Λ_∞_* is the limit of the conductivity at infinite high temperatures directly related to the number of charge carriers [38], *E_a_* the activation energy for charge transport, *R* is the universal gas constant, and *T_0_* the Vogel temperature. In complete analogy to the values of *Λ*, a larger value of *Λ_∞_* is also obtained for the TRS bearing the large [NTf_2_]^−^ anion (cf. Table 2), indicating that more ions contribute to the ion conductivity, i.e., this TRS might have a larger ionicity *I* as compared to [DMTR][OTf]. However, the molar ion conductivity of ILs does not only depend on *I*, but it is also related to the diffusion coefficients of the cations *D^+^* and anions *D^−^*, as revealed by the Nernst–Einstein (NE) relation [21,22]:

(2) $$\Lambda =I\;\left(D^{+}+D^{-}\right)\;\frac{F^{2}}{RT}=I\;\Lambda_{NE}$$

where *F* is the Faraday constant. Alternatively, the ionicity *I* can also be replaced by the so-called Haven ratio *H_R_*, which is defined as its reciprocal value (*H_R_ = I^−1^*) [39].

**Figure 4:**
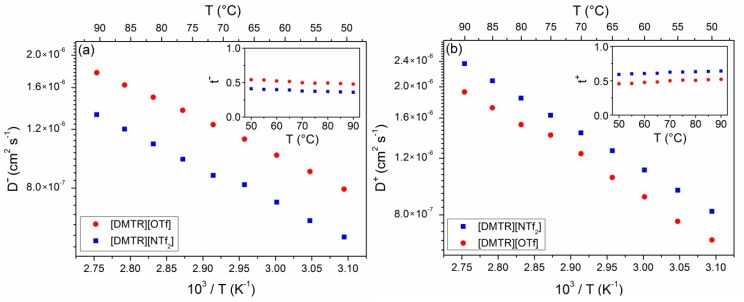
Diffusion coefficients of the (**a**) anions and (**b**) cations of [DMTR][OTf] and [DMTR][NTf_2_] as functions of the inverse temperature. The insets show the transference numbers of the two differently charged ionic species.

We performed ^1^H and ^19^F pulsed field gradient (PFG) NMR spectroscopy in order to obtain the diffusion coefficients *D* of the cations and anions as a measure for the ion mobility. The echo intensity was measured as a function of the applied gradient strength *g* with constant diffusion and gradient times (*Δ* and *δ*, respectively). According to the standard method described by Stejskal and Tanner [40], the values of *D* can directly be obtained from the slope of the logarithmic plot of the normalized echo intensities as a function of the diffusion function (*γ^2^δ^2^g^2^* × (*Δ−δ/3*), where *γ* is the gyromagnetic ratio), as depicted in Figures S7–S10. While the diffusion coefficient of the fluorine containing anions *D^−^* is directly obtained by analysis of the ^19^F PFG NMR spectra, the ^1^H PFG NMR data yield the diffusion coefficient of the cation *D^+^*, since this species contains the only protons. Thus, Figure 4 shows the values of *D^−^* and *D^+^* of [DMTR][OTf] and [DMTR][NTf_2_] in the temperature range of 50 °C ≤ *T* ≤ 90 °C.

The anion diffusion coefficients of [DMTR][OTf] are larger than the respective values of [DMTR][NTf_2_] at all measured temperatures (cf. Figure 4a), which is easily explainable by the different size of the anions. The larger [NTf_2_]^−^ anions diffuse slower than the [OTf]^−^ anions with a smaller ion volume. The different size of the anions also induces a different charge density (number of charges per volume unit), which further contributes to the diffusion behavior of the negatively charged species. However, the diffusion coefficients of the [DMTR]^+^ cations of the two TRSs behave differently (see Figure 4b). Despite their identical ion size, the cations diffuse slightly slower in [DMTR][OTf] compared to the cations in [DMTR][NTf_2_], indicating that the size of the anions also affects the values of *D^+^*. While the triflate anion and the [DMTR]^+^ cation have approximately the same ion volumes, the transference numbers of the anion *t^−^* and cation *t^+^* calculated from the diffusion coefficients (*t^±^ = D^±^*/(*D^+^ + D^−^*), see the insets of Figure 4a,b) are both close to 0.5. In contrast, the [NTf_2_]^−^ anion is about two times larger, and thus, it also has a lower transference number than the [DMTR]^+^ cation.

**Figure 5:**
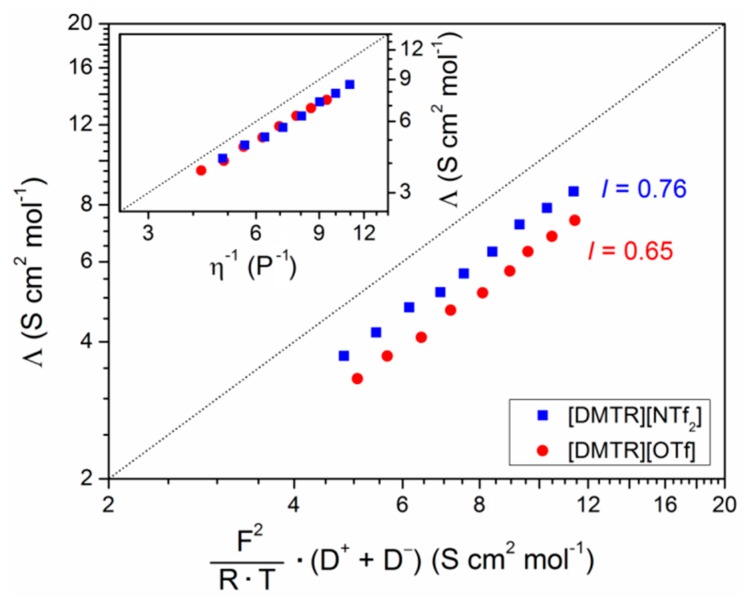
Molar ion conductivities of [DMTR][OTf] and [DMTR][NTf_2_] as a function of the calculated molar ion conductivity from the diffusion coefficients according to Equation (2). The dotted line indicates the ideal behavior where *Λ* = *Λ_NE_*. The inset shows the Walden plot (molar ion conductivities versus the inverse viscosity) of [DMTR][OTf] and [DMTR][NTf_2_].

In order to calculate the ionicities, the measured molar ion conductivity is plotted as function of the calculated conductivity values from diffusion coefficients (*Λ_NE_ = F^2^ ×* (*D^+^ + D^−^*)/(*R × T*)) according to the Nernst-Einstein equation (Equation (2)) in Figure 5. Obviously, there is a deviation from the ideal behavior (*Λ = Λ_NE_*, dotted line in Figure 5), i.e., the ionicities of both TRSs are lower than one. This is in good agreement with the so-called Walden plot (inset of Figure 5) where the molar conductivity is plotted as function of the inverse viscosity (see Figure S14, for viscosity data), and a similar deviation is observed from the ideal behavior. A value of *I* = 0.76 is calculated for [DMTR][NTf_2_] at *T* = 50 °C according to Equation (2), and it does not strongly depend on the temperature, since the data points in Figure 5 are almost parallel to the ideal line where *Λ* = *Λ_NE_* (see also Figure S15). This calculated value is in good agreement with the ionicity of the 1,3-dimethylimidazolium analogue (*I* = 0.76 at 30 °C, see also Table 2) [21] and it is significantly higher than the ionicities of TRSs bearing also a [NTf_2_]^−^ anion having longer alkyl chains as substituents on the 1,2,3-triazolium cation (0.53 ≤ *I* ≤ 0.63) [25]. It should be mentioned that the TRSs reported in ref. [25] are 1,3,4-trisubstituted derivatives since they were synthesized via CuAAC. However, the ionicity of [DMTR][OTf] is slightly lower (*I* = 0.65) than the TRSs, with the [NTf_2_]^−^ anion indicating that the structure of the anion has a significant influence on the number of ions contributing in TRSs to the conductivity. Because of the larger size of the anion, [DMTR][NTf_2_] has a lower charge density (number of charges per volume unit) as compared to [DMTR][OTf]. Thus, the intermolecular dispersion and repulsion forces, as well as the Coulomb interactions, are weaker in TRS bearing the [NTf_2_]^−^ anion, which reduces the formation of the aggregates and the charge transfer [22,23], and more ions are able to contribute actively to the conductivity in [DMTR][NTf_2_], as compared to [DMTR][OTf].

## 4. Conclusions

In conclusion, we synthesized three 1,3-dimethyl-1,2,3-triazolium salts with fluorine containing anions by direct methylation of 1,2,3-triazole which neither needed highly hazardous methyl azide nor required difficult removal of copper catalyst as required for the commonly used CuAAC. An in-depth understanding of the relationship between the structure and the ion transport properties of the 1,2,3-triazolium salts under investigation was obtained. The molar ion conductivities and ionicities of the 1,3-dimethyl-1,2,3-triazolium salts under investigation were enhanced in comparison to other 1,2,3-triazolium salts with longer alkyl substituents. However, the physicochemical properties such as ion conductivity, diffusion coefficients, densities, viscosities, and ionicities depended strongly on the molecular structure of the anion, which opens new pathways for the design of new materials with tuneable properties for a wide range of applications in electrochemical devices.

## Figures and Tables

**Table 1:** Structures of the 1,3-dimethyl-1,2,3-triazolium salts under investigation, their ion volumes (*V^+^* and *V^−^*), melting temperatures (*T_m_*), decomposition temperatures at 10% weight loss (*T_d10_*), and densities (*ρ*) at *T* = 50 °C.

Triazolium Salt	Cation	Anion	*V^+^* (Å^3^)	*V^−^* (Å^3^)	*T_m_* (°C)	*T_d10_* (°C)	*ρ* (g cm^−3^)
[DMTR][F]	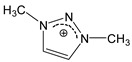	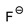	154 *^1^*	10 *^2^*	--- *^5^*	155	---
[DMTR][OTf]	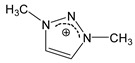	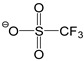	154 *^1^*	129 *^3^*	49.0	360	1.467
[DMTR][NTf_2_]	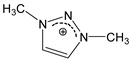	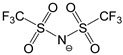	154 *^1^*	248 *^4^*	46.1	385	1.583

*^1^* Value for the structure analogous 1,3-dimethylimidazolium cation, taken from [35]. *^2^* Taken from [34]. *^3^* Taken from [35]. *^4^* Taken from [36]. *^5^* [DMTR][F] is crystalline, but it decomposes prior to melting; see Figure S11.

**Table 2:** Molar ion conductivities (*Λ*) and ionicities (*I*) at *T* = 50 °C, as well as VFT fit parameters (*Λ_∞_*, *E_a_* and *T_0_*) of the 1,3-dimethyl-1,2,3-triazolium salts under investigation. The corresponding values for the structure analogous 1,3-dimethylimidazolium bis(trifluoromethane sulfon)imide ([DMIM][NTf_2_]) are also included for comparison.

Ionic Liquid	*Λ* (S cm^2^ mol^−1^)	*I*	*Λ_∞_* (S cm^2^ mol^−1^)	*E_a_* (kJ mol^−1^)	*T_0_* (K)
[DMTR][OTf]	3.3	0.65	172	5.06	169
[DMTR][NTf_2_]	3.7	0.76	264	5.74	162
[DMIM][NTf_2_] *^1^*	2.5	0.76	---	---	---

*^1^* Values at 30 °C, taken from ref. [21].

## Supplementary Materials

The following are available online at http://www.mdpi.com/1996-1944/11/9/1723/s1, Figure S1. ^1^H NMR spectrum of [DMTR][F] in CD_3_OD (43 MHz, 28 °C), Figure S2. ^19^F NMR spectrum of [DMTR][F] in CD_3_OD (41 MHz, 28 °C), Figure S3. ^1^H NMR spectrum of [DMTR][OTf] in CD_3_OD (43 MHz, 28 °C), Figure S4. ^19^F NMR spectrum of [DMTR][OTf] in CD_3_OD (41 MHz, 28 °C), Figure S5. ^1^H NMR spectrum of [DMTR][NTf_2_] in CD_3_OD (43 MHz, 28 °C), Figure S6. ^19^F NMR spectrum of [DMTR][NTf_2_] in CD_3_OD (41 MHz, 28 °C), Figure S7. Normalized ^1^H echo intensities vs diffusion function of [DMTR][OTf] in the temperature range 50 °C ≤ *T* ≤ 90 °C. The slopes of the lines define the diffusion coefficients, Figure S8. Normalized ^19^F echo intensities vs diffusion function of [DMTR][OTf] in the temperature range 50 °C ≤ *T* ≤ 90 °C. The slopes of the lines define the diffusion coefficients, Figure S9. Normalized ^1^H echo intensities vs diffusion function of [DMTR][NTf_2_] in the temperature range 50 °C ≤ *T* ≤ 90 °C. The slopes of the lines define the diffusion coefficients, Figure S10. Normalized ^19^F echo intensities vs diffusion function of [DMTR][NTf_2_] in the temperature range 50 °C ≤ *T* ≤ 90 °C. The slopes of the lines define the diffusion coefficients, Figure S11. TGA curves of the three 1,3-dimethyl-1,2,3-triazolium salts under investigation, recorded with a heating rate of 5 K min^−1^, Figure S12. DSC heating traces of [DMTR][OTf] and [DMTR][NTf_2_], recorded with a heating rate of 5 K min^−1^. [DMTR][F] does not melt before complete decomposition, Figure S13. Densities of [DMTR][OTf] and [DMTR][NTf_2_] at different temperatures. The error bars are comparable to the size of the symbols, Figure S14. Viscosities of [DMTR][OTf] and [DMTR][NTf_2_] at different temperatures, Figure S15. Ionicities of [DMTR][OTf] and [DMTR][NTf_2_] at different temperatures.
