# Supplementary material for: Ion Transport Properties and Ionicity of 1,3-Dimethyl-1,2,3-Triazolium Salts with Fluorinated Anions
Source: Materials (Basel). 2018 Sep 14;11(9):1723. doi: 10.3390/ma11091723 (PMC6164264; doi:10.3390/ma11091723)
Supplement: Supplementary file 1 [file materials-11-01723-s001.pdf]

Supplementary Materials for

Ion Transport Properties and Ionicity of 1,3-Dimethyl-1,2,3-Triazolium Salts with Fluorinated Anions

Martin Pulst, Yury Golitsyn, Detlef Reichert and Jörg Kressler*

Faculty of Natural Sciences II, Martin Luther University Halle-Wittenberg, D-06099 Halle (Saale), Germany

Syntheses & Characterization

1-Methyl-1,2,3-triazole was synthesized according to established literature procedures[1–3] and the 1,3-dimethyl-1,2,3-triazolium salts were prepared according to the modified synthesis procedures of similar ionic liquids.[4–8]

Syntheses of 1-Methyl-1,2,3-triazole (MeTR)

- Route A: Direct Synthesis of MeTR from 1,2,3-Triazole

  1,2,3-Triazole (50 g; 0.72 mol) was dissolved in a suspension of THF (750 mL) and potassium carbonate (200 g; 1.45 mol). Methyl iodide (155 g; 1.09 mol) was added and the reaction mixture was stirred vigorously at room temperature for 4 hr. The yellowish solution was filtrated and the solvent removed under vacuum. 1-Methyl-1,2,3-triazole was purified as a yellow oil with 60 % yield.

- Route B: Synthesis of MeTR from Lithium 1,2,3-Triazolate

  Lithium 1,2,3-triazolate (0.75 g; 1.0 mmol; synthesized from 1,2,3-triazole as reported in our previous publication[9]) was dissolved in methanol (5 mL). Methyl iodide (1.41 g; 1.0 mmol) was added and the reaction mixture was stirred at 60 °C for 24 h. The solvent was removed under vacuum and the product was extracted with methylene chloride (3 × 10 ml) after addition of water (10 mL). 1-Methyl-1,2,3-triazole was purified as a yellow oil with 30 % yield.

^{1}H NMR (43 MHz, $CDCl_3$, 28°C): δ (ppm) = 7.67 (s, 1H); 7.54 (s, 1H); 4.10 (s, 3H).

Synthesis of 1,3-Dimethyl-1,2,3-triazolium iodide ([DMTR][I])

A mixture of 1-methyl-1,2,3-triazole (5.00 g; 60.3 mmol) and methyl iodide (12.84 g; 90.4 mmol) was heated to T = 60 °C and stirred for 24 h. After removal of the excess of methyl iodide and drying in vacuum (≈ 400 μbar), yellow crystals of 1,3-dimethyl-1,2,3-triazolium iodide were purified with a quantitative yield.
^{1}H NMR (43 MHz, CD_3OD, 28°C): δ (ppm) = 8.81 (s, 2H); 4.47 (s, 6H, $^1J_{C\text{-}H}$ = 145.8 Hz)

Synthesis of 1,3-Dimethyl-1,2,3-triazolium fluoride ([DMTR][F])

1,3-Dimethyl-1,2,3-triazolium iodide (3.21 g; 14.3 mmol) dissolved in water (10 mL) was added dropwise to a solution of silver fluoride (1.81 g; 14.3 mmol) and water (10 mL). The mixture was stirred for 1 hr with exclusion of light. After filtration, the solvent was removed in vacuum and the crude residue was added into dimethyl sulfoxide (5 mL). The precipitation was filtered and dried in vacuum (≈ 400 μbar) to obtain the colorless crystals of 1,3-dimethyl-1,2,3-triazolium fluoride in 90 % yield.
^{1}H NMR (43 MHz, CD_3OD, 28°C): δ (ppm) = 8.71 (s, 2H); 4.37 (s, 6H, $^1J_{C\text{-}H}$ = 146.6 Hz)
^{13}C NMR (100 MHz, CD_3OD, 28°C): δ (ppm) = 132.4; 40.4
^{19}F NMR (41 MHz, CD_3OD, 28°C): δ (ppm) = – 150.1

Synthesis of 1,3-Dimethyl-1,2,3-triazolium triflate ([DMTR][OTf])

A mixture of 1-methyl-1,2,3-triazole (2.00 g; 24.1 mmol) and methyl trifluoromethanesulfonate (3.96 g; 24.1 mmol) was heated to $T = 60$ °C and stirred for 24 h. After drying in vacuum (≈ 400 µbar), ivory crystals of 1,3-dimethyl-1,2,3-triazolium triflate were purified with a quantitative yield.

^{1}H NMR (43 MHz, CD_3OD, 28°C): δ (ppm) = 8.67 (s, 2H); 4.38 (s, 6H, $^1J_{C\text{-}H}$ = 145.9 Hz)
^{13}C NMR (125 MHz, CD_3OD, 28°C): 132.4; 122.8 (q, $^1J_{C\text{-}F}$ = 318.6 Hz); 40.4
^{19}F NMR (41 MHz, CD_3OD, 28°C): δ (ppm) = – 78.8

Synthesis of 1,3-Dimethyl-1,2,3-triazolium bis((trifluoromethyl)sulfonyl)imide ([DMTR][NTf_2])

A mixture of 1-methyl-1,2,3-triazole (1.00 g; 12.1 mmol) and *N*-methyl bis((trifluoromethyl)sulfonyl)imide (3.56 g; 12.1 mmol) was heated to $T = 60$ °C and stirred for 24 h. After drying in vacuum (≈ 400 µbar), colorless crystals of 1,3-dimethyl-1,2,3-triazolium bis((trifluoromethyl)sulfonyl)imide were received in quantitative yield.

^{1}H NMR (43 MHz, CD_3OD, 28°C): δ (ppm) = 8.66 (s, 2H); 4.38 (s, 6H, $^1J_{C\text{-}H}$ = 146.3 Hz)
^{13}C NMR (125 MHz, CD_3OD, 28°C): 132.4; 122.8 (q, $^1J_{C\text{-}F}$ = 320.4 Hz); 40.4
^{19}F NMR (41 MHz, CD_3OD, 28°C): δ (ppm) = – 79.4

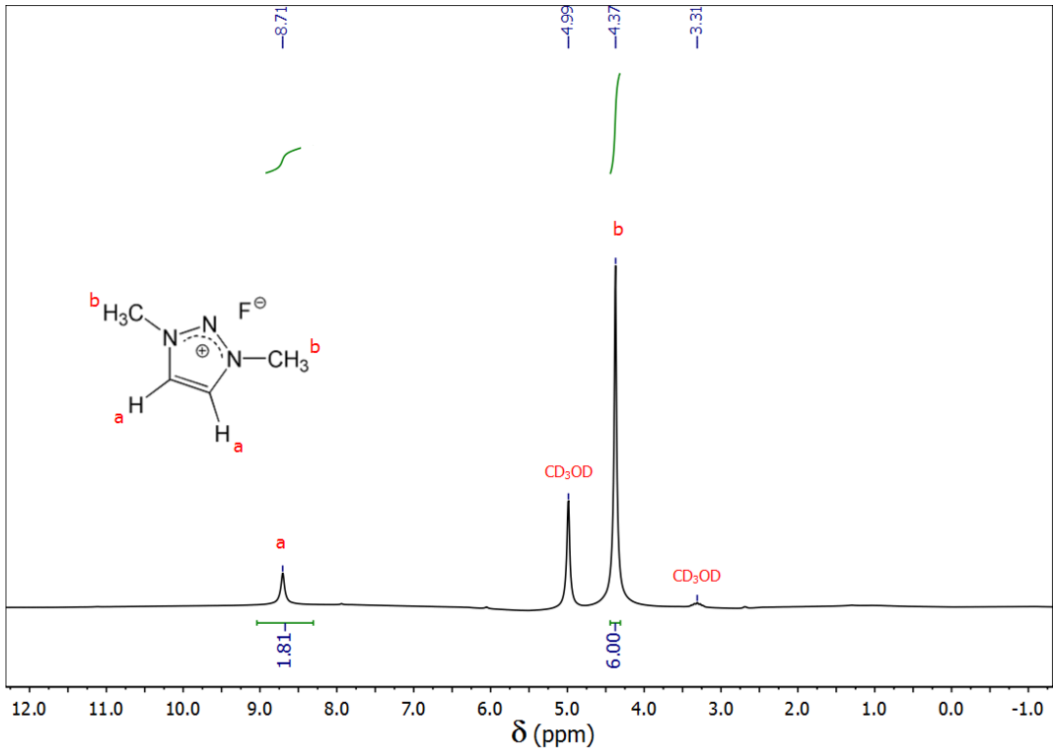

Figure S1. ^{1}H NMR spectrum of [DMTR][F] in CD_3OD (43 MHz, 28 °C).

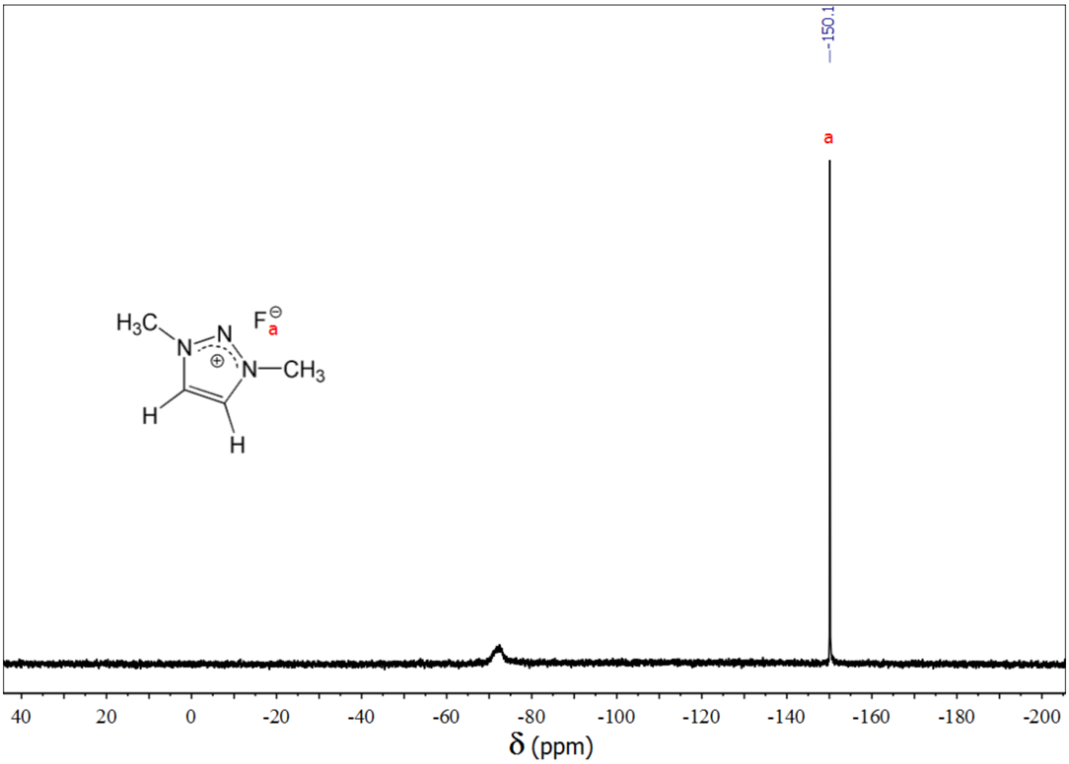

Figure S2. ^{19}F NMR spectrum of [DMTR][F] in CD_3OD (41 MHz, 28 °C).

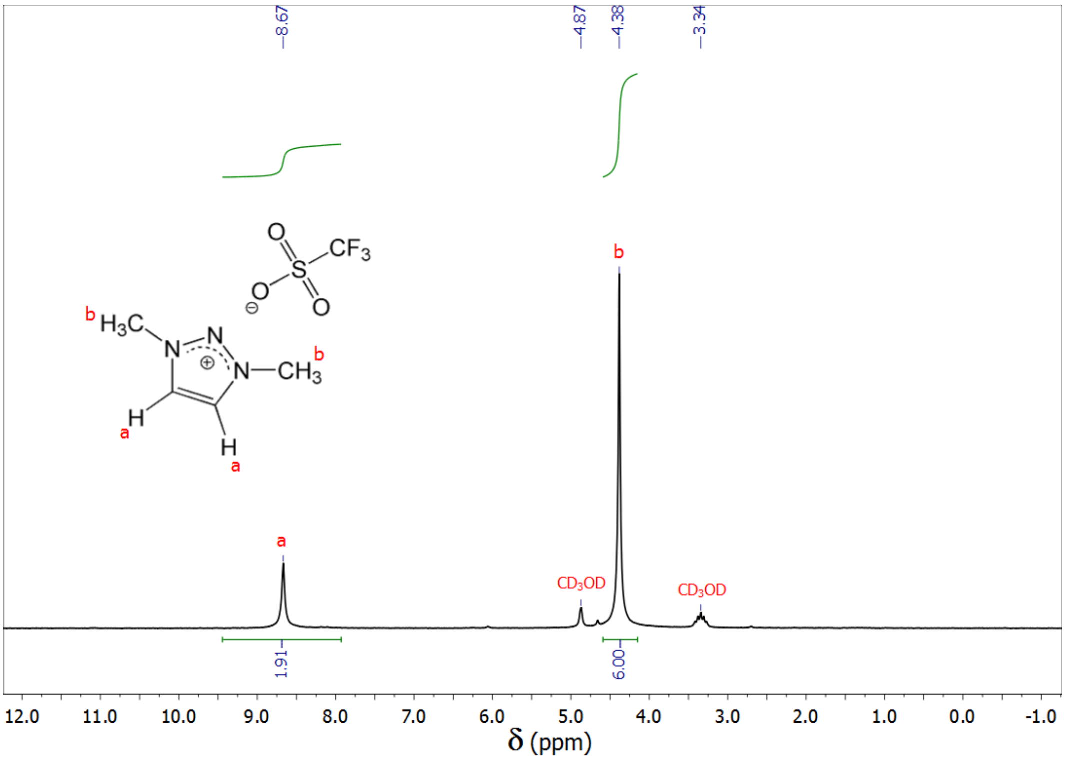

Figure S3. ^{1}H NMR spectrum of [DMTR][OTf] in CD_3OD (43 MHz, 28 °C).

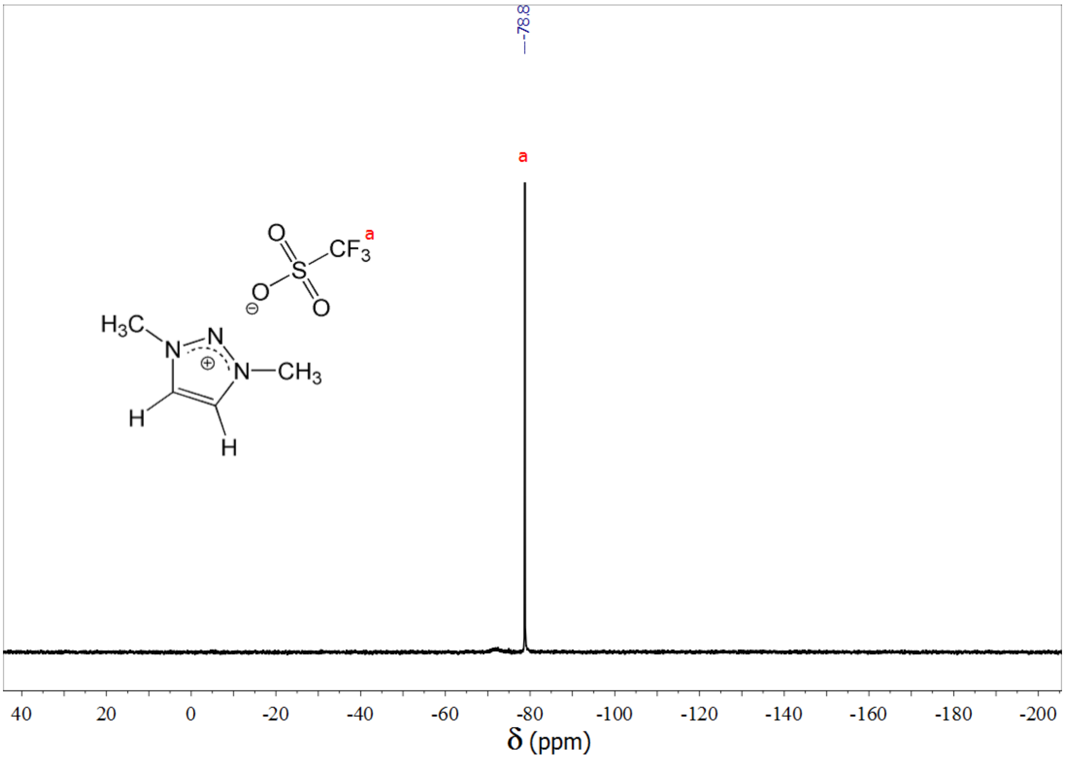

Figure S4. ^{19}F NMR spectrum of [DMTR][OTf] in CD_3OD (41 MHz, 28 °C).

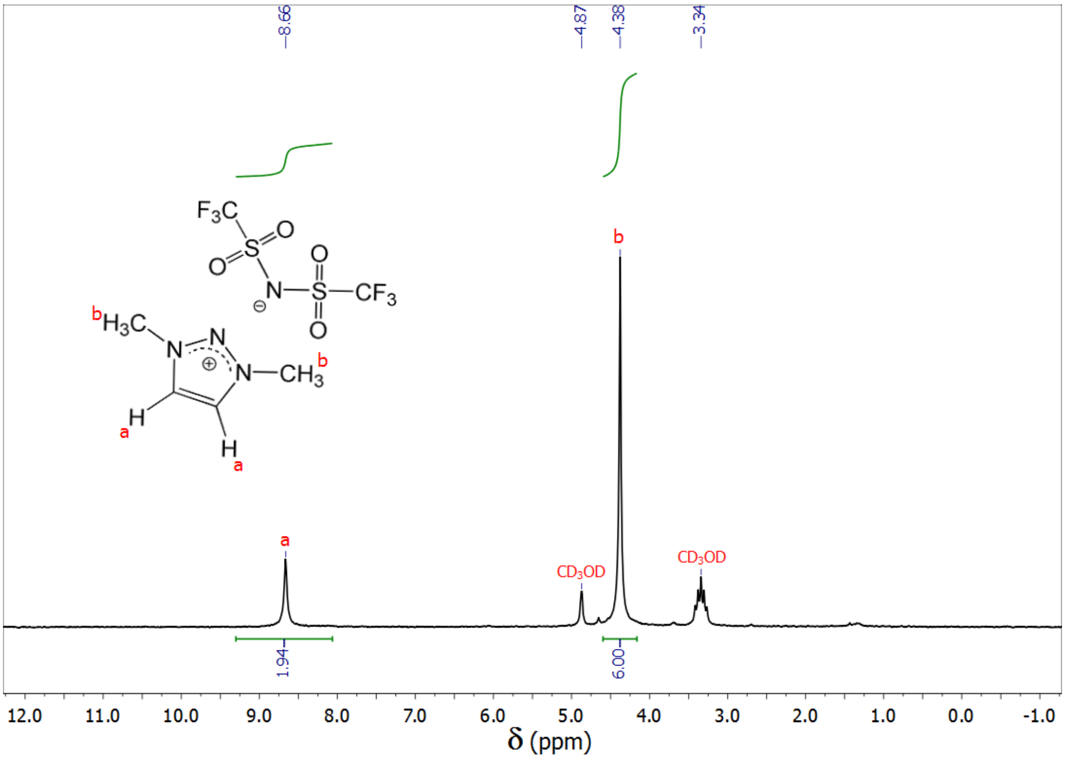

Figure S5. ^{1}H NMR spectrum of [DMTR][NTf_2] in CD_3OD (43 MHz, 28 °C).

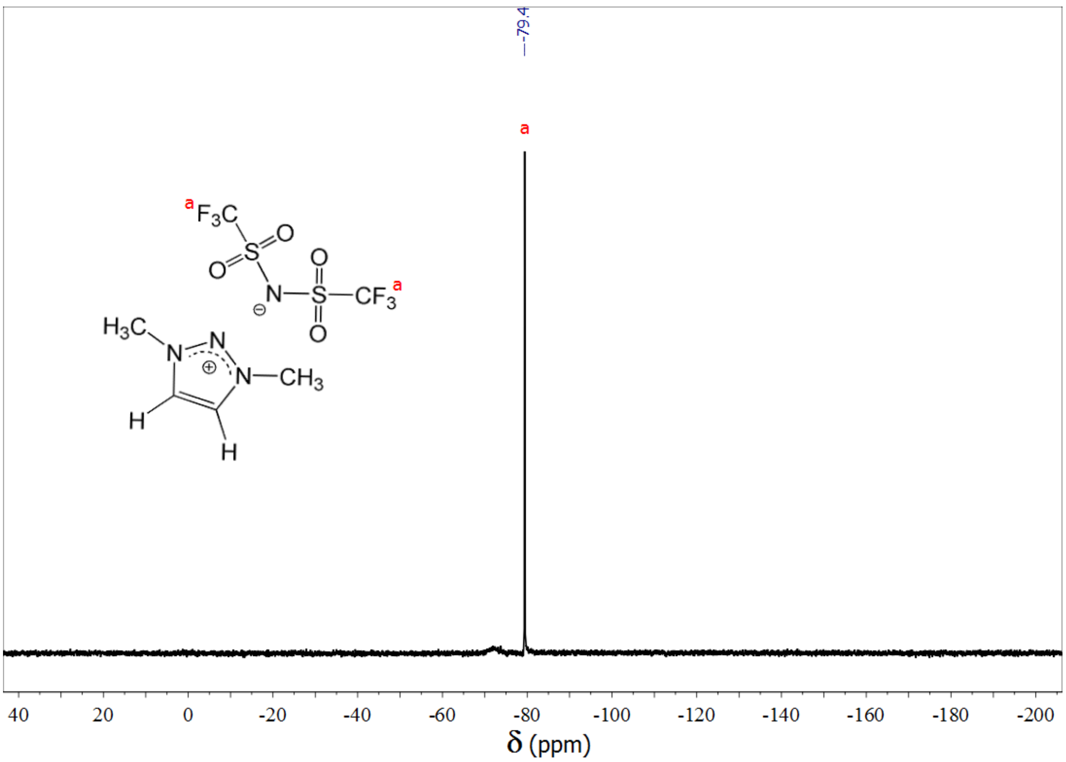

Figure S6. ^{19}F NMR spectrum of [DMTR][NTf_2] in CD_3OD (41 MHz, 28 °C).

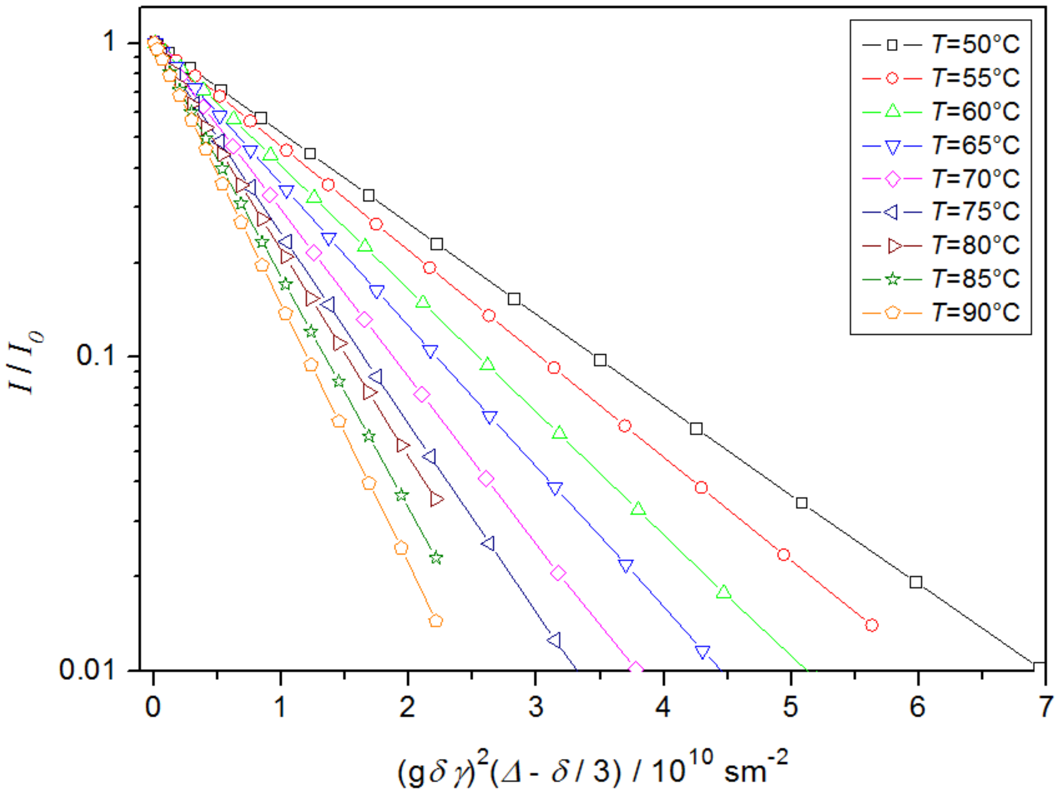

Figure S7. Normalized ^{1}H echo intensities vs diffusion function of [DMTR][OTf] in the temperature range 50 °C ≤ T ≤ 90 °C. The slopes of the lines define the diffusion coefficients.

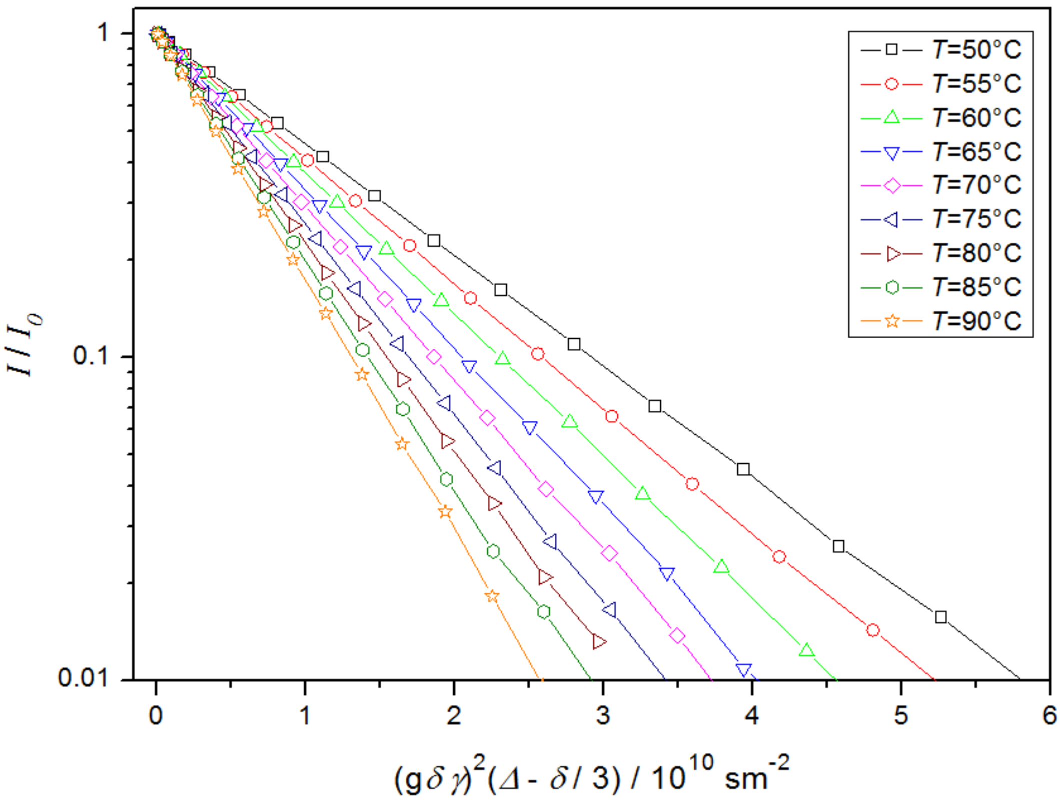

Figure S8. Normalized ^{19}F echo intensities vs diffusion function of [DMTR][OTf] in the temperature range 50 °C ≤ T ≤ 90 °C. The slopes of the lines define the diffusion coefficients.

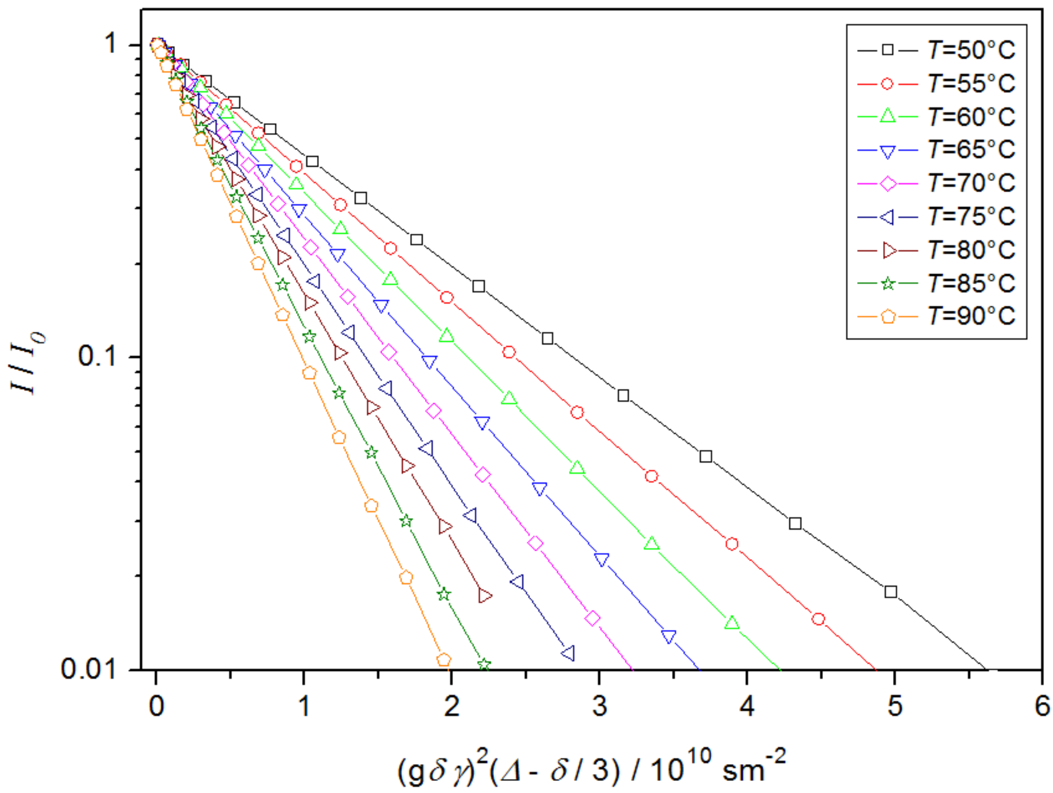

Figure S9. Normalized ^{1}H echo intensities vs diffusion function of [DMTR][NTf_2] in the temperature range 50 °C ≤ T ≤ 90 °C. The slopes of the lines define the diffusion coefficients.

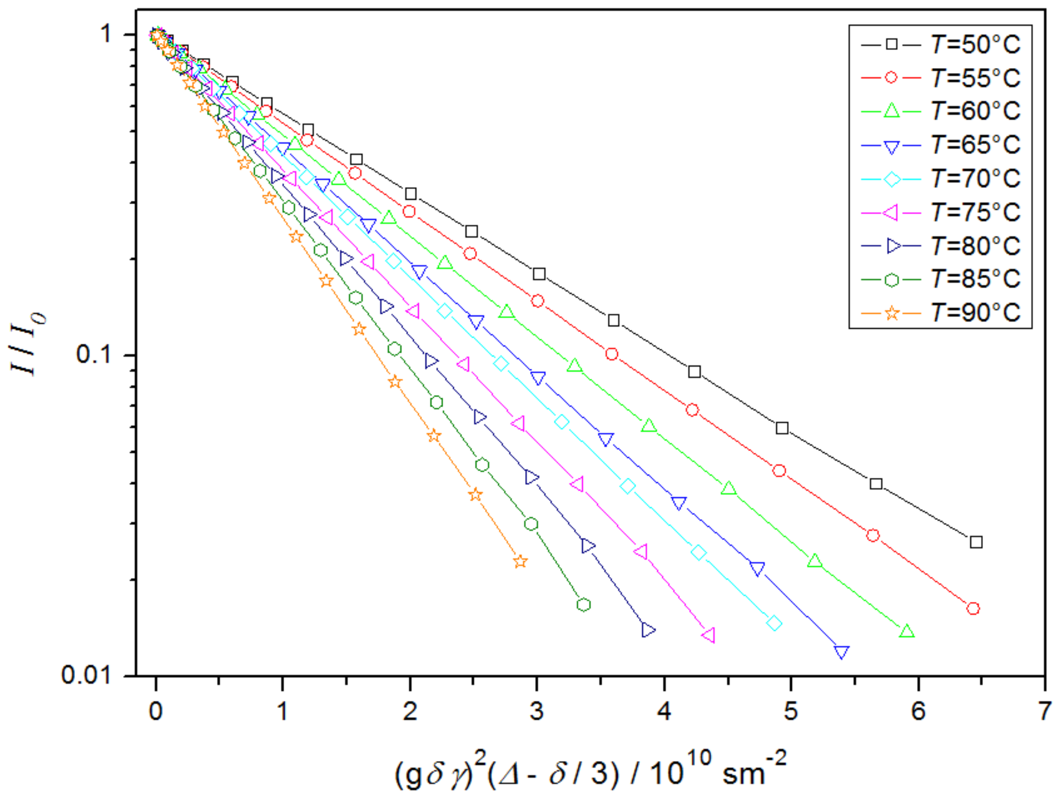

Figure S10. Normalized ^{19}F echo intensities vs diffusion function of [DMTR][NTf$_2$] in the temperature range 50 °C ≤ T ≤ 90 °C. The slopes of the lines define the diffusion coefficients.

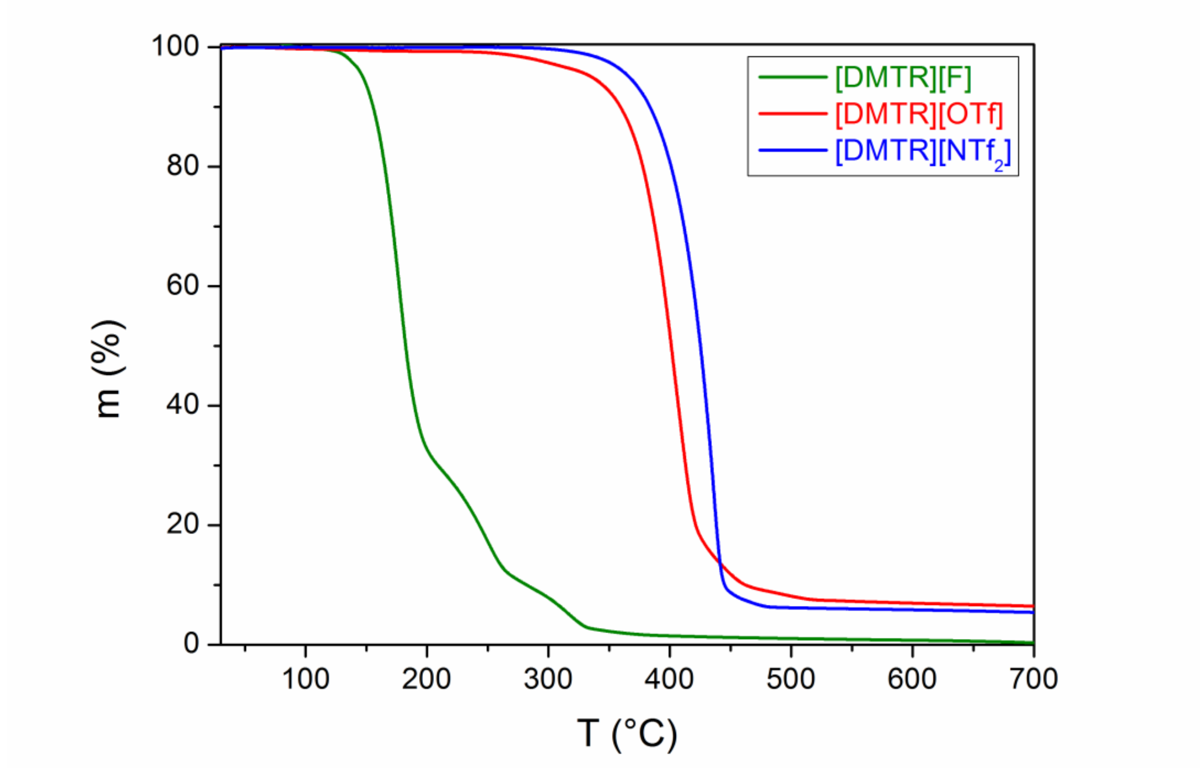

Figure S11. TGA curves of the three 1,3-dimethyl-1,2,3-triazolium salts under investigation recorded with a heating rate of 5 K min^{-1}.

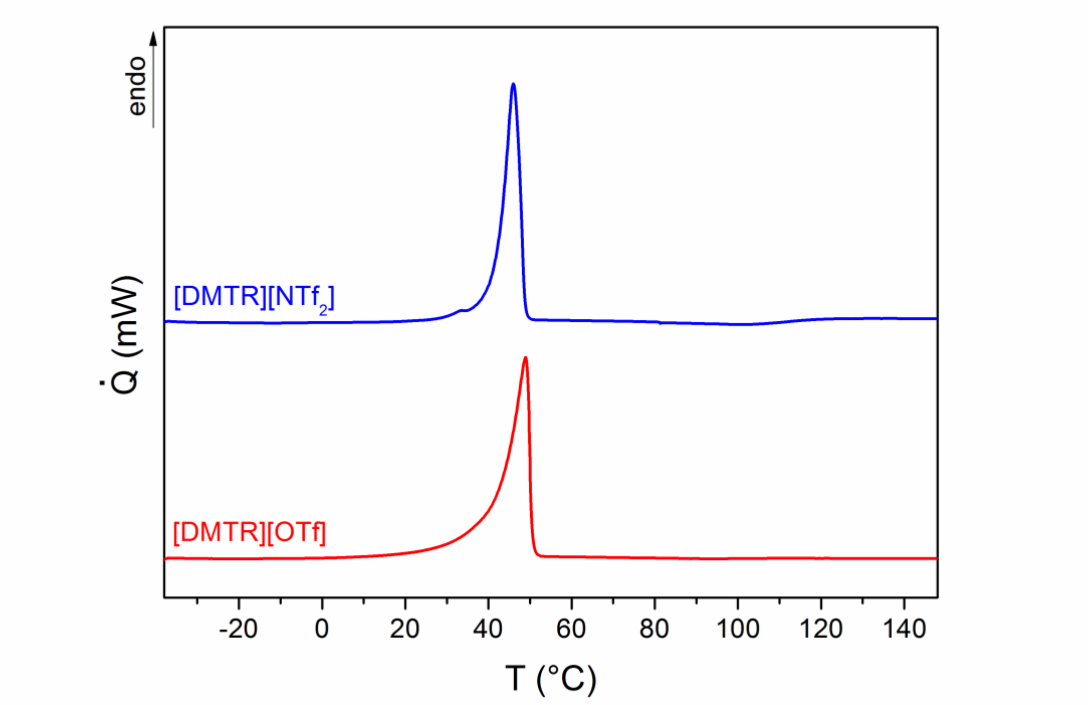

Figure S12. DSC heating traces of [DMTR][OTf] and [DMTR][NTf$_2$] recorded with a heating rate of 5 K min^{-1}. [DMTR][F] does not melt before complete decomposition.

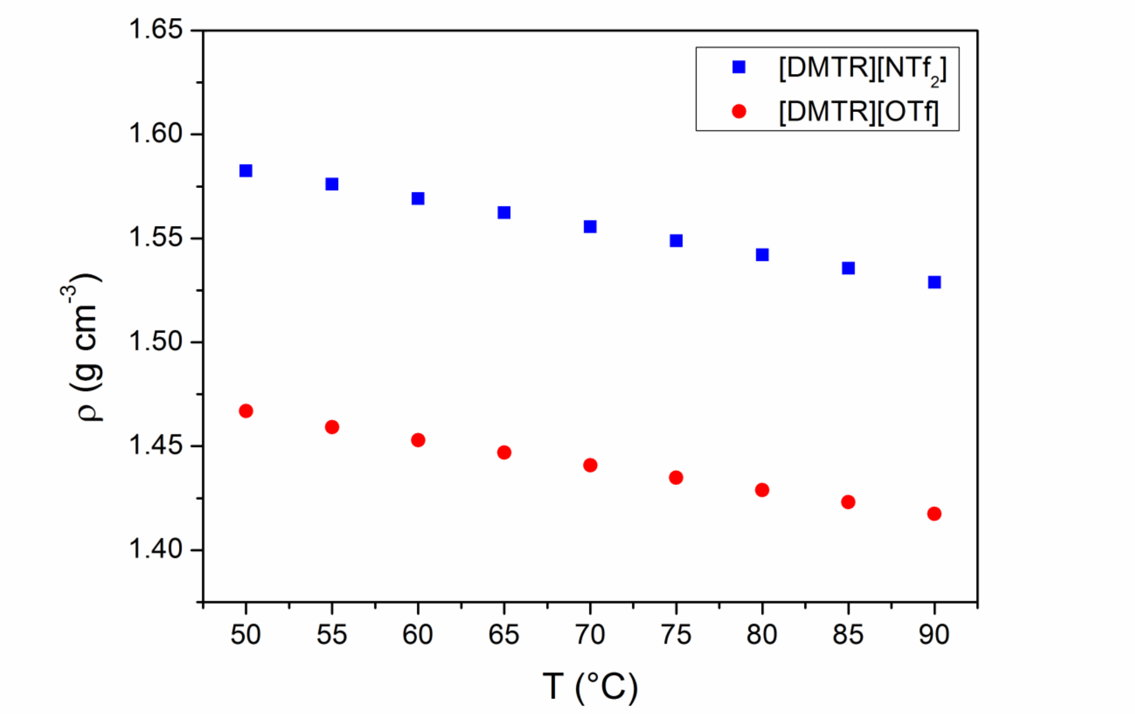

Figure S13. Densities of [DMTR][OTf] and [DMTR][NTf$_2$] at different temperatures. The error bars are comparable to the size of the symbols.

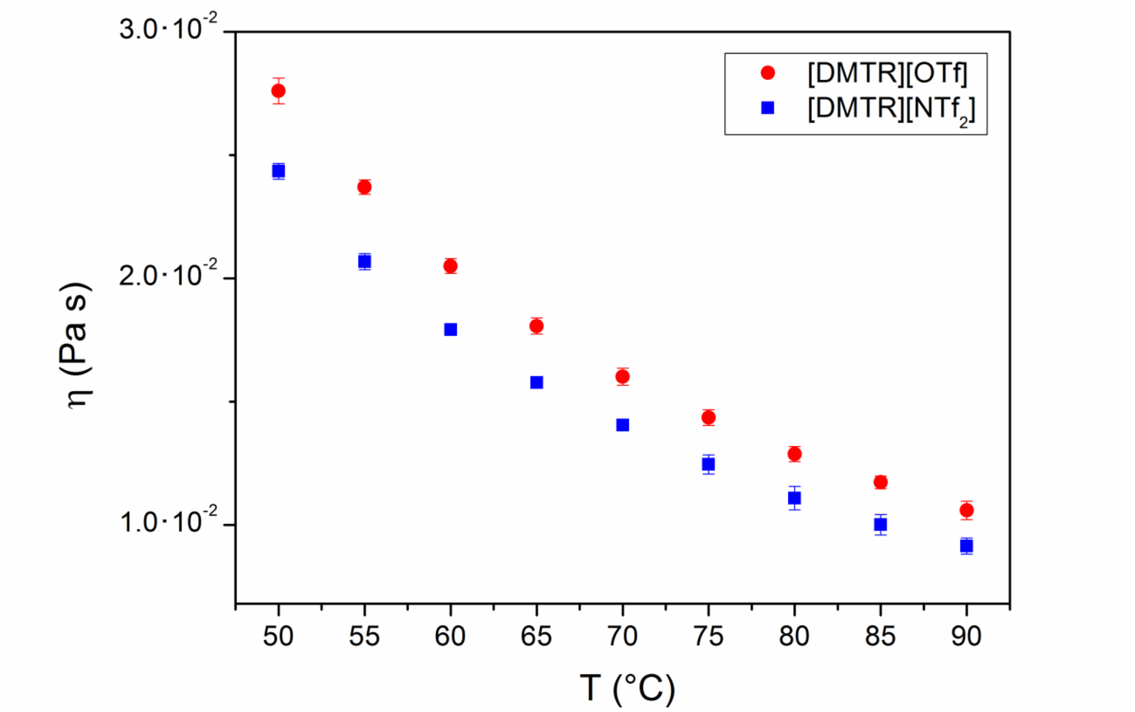

Figure S14. Viscosities of [DMTR][OTf] and [DMTR][NTf_2] at different temperatures.

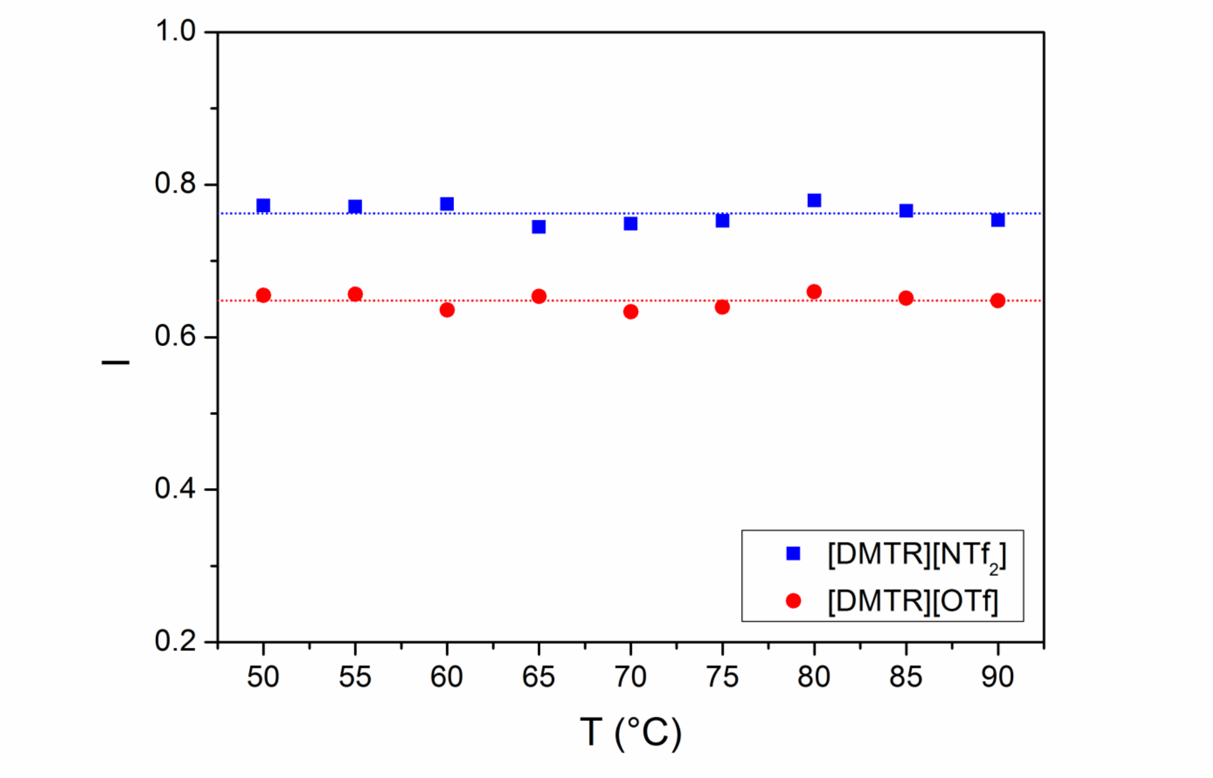

Figure S15. Ionicities of [DMTR][OTf] and [DMTR][NTf_2] at different temperatures. The horizontal lines are used as a guide.

References

(1) Seefeld, M. A.; Rouse, M. B.; Heerding, D. A.; Peace, S.; Yamashita, D. S.; Mcnulty, K. C. Inhibitors of Akt Activity. WO2008/098104 A1, 2008.

(2) Stappert, K.; Ünal, D.; Spielberg, E. T.; Mudring, A.-V. Influence of the Counteranion on the Ability of 1‑Dodecyl-3- methyltriazolium Ionic Liquids to Form Mesophases. *Cryst. Growth Des.* **2015**, *15*, 752–758.

(3) Bagal, S. K.; Bungay, P. J.; Denton, S. M.; Gibson, K. R.; Glossop, M. S.; Hay, T. L.; Kemp, M. I.; Lane, C. A. L.; Lewis, M. L.; Maw, G. N.; et al. Discovery and Optimization of Selective Nav1.8 Modulator Series That Demonstrate Efficacy in Preclinical Models of Pain. *ACS Med. Chem. Lett.* **2015**, *6*, 650–654.

(4) Sanghi, S.; Willett, E.; Versek, C.; Tuominen, M.; Coughlin, E. B. Physicochemical Properties of 1,2,3-Triazolium Ionic Liquids. *RSC Adv.* **2012**, *2*, 848–853.

(5) Begtrup, M.; Kristensen, P. A. Reactions between Azolium Salts and Nucleophilic Reagents I. Bromo-1,2,3-Triazolium Salts as Brominating Reagents. *Acta Chem. Scand.* **1969**, *23*, 2733–2740.

(6) Begtrup, M. Reactions between Azolium Salts and Nucleophilic Reagents II. Bromo-1,2,3-Triazolium Salts and Sodium Hydroxide. *Acta Chem. Scand.* **1971**, *25*, 249–259.

(7) Zhu, Z.-Q.; Jiang, M.-Y.; Zheng, C.-G.; Xiao, J.-C. Efficient Synthesis of 1-Alkyl-3-methylimidazolium Fluorides and Possibility of the Existence of Hydrogen Bonding between Fluoride Anion and C(sp^3)–H. *J. Fluor. Chem.* **2012**, *133*, 160–162.

(8) Sarmiento, G. P.; Zelcer, A.; Espinosa, M. S.; Babay, P. A.; Mirenda, M. Photochemistry of Imidazolium Cations. Water Addition to Methylimidazolium Ring Induced by UV Radiation in Aqueous Solution. *J. Photochem. Photobiol. A Chem.* **2016**, *314*, 155–163.

(9) Pulst, M.; Elgabarty, H.; Sebastiani, D.; Kressler, J. The Annular Tautomerism of Lithium 1,2,3-Triazolate. *New J. Chem.* **2017**, *41*, 1430–1435.
